# Study of primary immunodeficiencies in Algeria

**Published:** 2011-01-10

**Authors:** Nadia Kechout, Nabila Attal, Fetouma Doudou, Rachida Boukari, Mohamed Ardjoun, Mohamed Cherif Abbadi

**Affiliations:** 1 Department of Immunology, Institut Pasteur d’Algérie, Algiers, Algeria; 2 Department of Pediatrics, CHU Blida, Algeria; 3 Blood Transfusion of Army, Algiers, Algeria

## 

Primary immunodeficiencies (PIDs) are heritable disorders of immune system function. These defects are rare, but seem to be more frequent in populations with high consanguinity. The defect may affect T cells, B cells, phagocyte cells or complement proteins. Patients have increased susceptibility to recurrent infections, and may suffer from, allergy, auto-immune disorders or cancers.

The assessment of 303 patients suspected to have a primary immunodeficiency during seven years (January 2003 to July 2010) allowed us to diagnose 75 PIDs aged between 3 months and 32 years: 51 (68%) males and 24 (32%) females; the sex ratio is 2.12. 40 % of the patients are the offspring of consanguineous marriages.

The immunological tests that we used include measurment of serum IgG, IgA, IgM, IgE levels and IgG subclass levels; tetrazolium nitroblue test; lymphocyte immunophenotyping and flow cytometry evaluation of surface proteins as: CD11, CD15, CD18, MHC class II, CD40, CD40 ligand, CD25.

We report in this study the results from the 75 patients: 13 have “severe combined immunodeficiency (SCID), 11: MHC II deficiency, 30: agammaglobulinaemia, 1: Hyper-IgM syndrome, 9: common variable immunodeficiency, 1: IgG2 deficiency, 9: phagocyte defects, and 1 ataxia-telangiectasia.

The molecular characterization of these deficiencies is important for diagnosis in some cases, for prenatal diagnosis and for potential therapeutic intervention strategies. We plan to set up other tests for evaluation of the other PIDs.

